# Unveiling the Complex Interplay: Sudden Emergence of First‐Rank Schneiderian Symptoms Following TMS in a Patient With Severe Depression and Complex Trauma

**DOI:** 10.1155/crps/5528976

**Published:** 2026-03-05

**Authors:** Jaskaran Singh, Luba Leontieva, S. D. Sperry, Karan Sachdeva, Sanobar Jaka

**Affiliations:** ^1^ Department of Psychiatry and Behavioral Sciences, Nassau University Medical Center, East Meadow, New York, USA, nuhealth.net; ^2^ Department of Psychiatry, SUNY Upstate Medical University, Syracuse, New York, USA, upstate.edu; ^3^ Department of Psychiatry, Teerthanker Mahaveer Medical College and Research Center, Moradabad, Uttar Pradesh, India, tmu.ac.in

## Abstract

Schizophrenia‐spectrum disorders are characterized by psychotic symptoms, including hallucinations, delusions, and disorganized thinking. Schneider’s first‐rank symptoms (FRS)—including thought broadcasting and experiences of external control—are clinically salient but not ergonomic and require careful differential diagnosis. The emergence of new‐onset psychosis in midlife, particularly in individuals without prior psychotic history, warrants a broad biopsychosocial evaluation. We present the case of a 47‐year‐old male with chronic major depressive disorder and complex developmental trauma who developed abrupt FRS‐like phenomena (prominently thought broadcasting) and auditory hallucinations following a period marked by (1) severe pneumonia with lung abscess, (2) escalating anxiety and depressive distress, (3) exposure to substances including reported synthetic cannabinoids (“Spice”) with a urine toxicology positive for amphetamines, and (4) neuromodulation via 20 sessions of transcranial magnetic stimulation (TMS). Concurrently, the patient experienced a severe psychosocial/legal stressor (investigation related to child sexual exploitation material), associated with profound shame and fear. Rather than attributing symptoms to a single trigger, this case highlights a convergence model—medical inflammation, substance toxicity, trauma‐related vulnerability, and acute legal stress—potentially disrupting cerebral homeostasis and precipitating psychosis and suicidality. Treatment included discontinuation of TMS, pharmacologic stabilization, and intensive psychotherapy, with partial remission of psychotic symptoms. There is a need for structured monitoring for emergent psychosis and multidimensional suicide risk in high‐risk patients receiving neuromodulation.

## 1. Introduction

Schizophrenia and related psychotic disorders are characterized by delusions, hallucinations, disorganized thinking, and functional impairment [1]. Schneider described first‐rank symptoms (FRS), such as thought broadcasting, voices commenting, and passivity experiences, as clinically striking phenomena associated with psychosis [2]. However, FRS can occur across diagnostic contexts (e.g., trauma‐related and obsessive‐compulsive spectrum presentations) and therefore must be interpreted within the whole clinical picture.

Transcranial magnetic stimulation (TMS) is an evidence‐based intervention for treatment‐resistant depression that uses magnetic pulses to modulate cortical excitability and distributed neural circuits [3, 4]. TMS is generally well tolerated, with common adverse effects including headache and scalp discomfort; rare serious events include seizures and treatment‐emergent mania [5–7]. Reports of TMS‐associated psychotic symptoms are uncommon but clinically important given increasing real‐world use [3].

Suicidality in psychosis is increasingly conceptualized as multidimensional, reflecting heterogeneous pathways (e.g., affective dysregulation, shame/defeat, command phenomena, and substance‐associated psychosis) with distinct psychological underpinnings and prevention needs [8, 9].In this report, we describe a patient with chronic depression and complex trauma who developed sudden FRS‐like symptoms and subsequent suicide attempts in the setting of intersecting biological, psychological, and social stressors, including TMS exposure.

## 2. Case Presentation

The patient is a 47‐year‐old Caucasian male, single, unemployed, receiving SSI, with a psychiatric history of recurrent major depressive disorder (onset age 13), anxiety, alcohol and cannabis use disorder (since adolescence), and an unspecified trauma‐ and stressor‐related disorder. He had no psychiatric hospitalizations prior to the onset of current psychotic symptoms and no known prior history of mania or psychosis. Medical history includes asthma and arthritis.

He was hospitalized twice within 2 weeks (May and June 2024) following two suicide attempts by overdose (amitriptyline and haloperidol). He described severe anxiety and psychological distress related to ego‐dystonic experiences of thought broadcasting and associated shame. He also reported auditory hallucinations consisting of derogatory comments and perceived environmental harassment (e.g., slamming doors, noises), contributing to distress and suicidality.

### 2.1. Family and Trauma‐Related History

The patient has a significant family history of psychiatric illness, including maternal depression, paternal depression with a prior suicide attempt and hoarding disorder, and suicide by hanging in a paternal grandfather during the patient’s adolescence. Both parents were disabled.

He reports a history of repeated sexual abuse by neighborhood peers between the ages of 5 and 13 years. Due to worsening depression and anxiety, he transitioned to homeschooling beginning in eighth grade but successfully completed high school. He briefly worked after graduation but was unable to sustain employment secondary to severe depressive symptoms. He has received Supplemental Security Income (SSI) since age 22.

The patient has never married and has no children and reports minimal social support, noting only one close friendship throughout his life. He lived with his family until his father’s death at age 45, after which he was left responsible for a cluttered home he was unable to maintain.

### 2.2. Substance Use History

The patient reported daily/near‐daily cannabis and alcohol use over ~30 years. He also reported use of synthetic cannabinoids (“Spice”) around the time of symptom emergence. Urine toxicology was positive for amphetamines at admission. The exact dose/frequency of synthetic cannabinoid exposure in the week preceding psychosis could not be reliably quantified from available records/history, limiting dose–response inference; future cases would benefit from structured quantification (amount, route, timing, product type, and potency).

### 2.3. Relevant Treatment History

He received long‐term outpatient psychiatric care (ages 13–40), including individual and family therapy and pharmacotherapy (amitriptyline and gabapentin with sustained benefit). After his long‐term psychiatrist retired, he experienced increased anxiety.

In September 2023, he was hospitalized for severe pneumonia with a right lung abscess. In November 2023, he disclosed involvement in an investigation related to child sexual exploitation material. In December 2023, due to worsening depressive symptoms, he underwent 20 sessions of TMS. He noted mild initial improvement, but by February 2024, he reported persistent intrusive thoughts, paranoia, and auditory hallucinations not previously documented. These symptoms were experienced as ego‐dystonic and intolerable, culminating in suicide attempts.

During psychiatric hospitalization, an SSRI and antipsychotic trials were initiated; multiple antipsychotic trials yielded limited benefit, and clozapine was trialed without response and with subjective worsening of intrusive thoughts at 50 mg. Lorazepam provided partial symptomatic relief of anxiety and sleep disturbance. He was discharged on fluvoxamine and olanzapine with outpatient follow‐up. A detailed psychiatric and psychosocial chronology is provided in Table 1.

**Table 1:** Psychiatric and psychosocial timeline.

Date/age	Event
Ages 5–13	Experienced sexual abuse by neighborhood boys.
Age 13	Onset of depressive symptoms; began outpatient psychiatric care.
Age 15	Initiated alcohol and cannabis use.
Age 22	Began receiving Supplemental Security Income.
Age 25	Mother passed away.
Age 40 (2017)	Transitioned to a new psychiatrist following the retirement of the prior provider.
Age 42 (2019)	Diagnosed with PTSD and anxiety; initiated treatment with trazodone and gabapentin.
Age 45 (2022)	Father passed away.
September 2023	Hospitalized for pneumonia complicated by a right lung abscess.
November 2023	Disclosed involvement in an ongoing child pornography investigation.
December 2023	Completed 20 sessions of transcranial magnetic stimulation for depression, with mild initial improvement.
February 2024	Reported persistent intrusive thoughts, paranoia, and auditory hallucinations following TMS treatment.
May 2024	First psychiatric hospitalization following a suicide attempt via amitriptyline overdose.
June 2024	Second psychiatric hospitalization following a suicide attempt via haloperidol overdose.

## 3. Discussion

### 3.1. Biopsychosocial Convergence and Disruption of Cerebral Homeostasis

This case is best conceptualized through a biopsychosocial convergence model rather than single‐cause attribution. The patient’s psychosis‐like symptoms likely reflect disruption of cerebral homeostasis arising from intersecting contributors:

Biological: systemic inflammation and physiologic stress after severe pneumonia/lung abscess; potential neuroinflammatory pathways increasing vulnerability to psychiatric decompensation [10, 11].
Substance‐related: reported synthetic cannabinoid exposure and amphetamine‐positive toxicology—both associated with acute psychiatric toxicity and capable of precipitating paranoia, hallucinations, and psychosis in individuals without prior psychotic disorders [12].
Psychological: complex developmental trauma, chronic depression/anxiety, ego‐dystonic intrusive phenomena, and limited coping capacity.
Social: marked isolation, disability and functional impairment, bereavement, and acute legal stress associated with intense shame and fear.

This relationship between treatment with TMS and the sudden onset of psychotic symptoms is still not well understood. A similar case study was found in which a 55‐year‐old male patient, diagnosed with major depressive disorder and generalized anxiety disorder without a history of psychosis or mania, experienced TMS‐induced predominant mania, including sleep disturbances, and delusional symptoms, including persecutory paranoia and grandiosity [13]. A case report on a 62‐year‐old Brazilian woman revealed the onset of manic psychosis after transcranial DC stimulation therapy [14]. Another case study revealed a sudden occurrence of psychotic symptoms (delusions) after repetitive transcranial magnetic stimulation (rTMS) [15]. A possible explanation for acute psychotic symptoms may lie in the pathophysiology of TMS, causing an increase in extracellular levels of both dopamine and glutamate [16, 17]. Although TMS seems to be a generally safer technique, with a benign profile of side effects, there is insufficient safety data in clinical populations, especially those suffering from major depression. In our case, treatment with TMS followed by the sudden onset of unspecified psychotic symptoms and auditory hallucinations suggests a possible adverse side effect. However, no study has found the sudden occurrence of intrusive thoughts leading to suicide attempts in a patient with chronic stable depression with a history of childhood trauma, making this case particularly noteworthy.

### 3.2. TMS: Benefits, Adverse Effects, and Plausibility of Rare Psychotic Reactions

TMS is generally safe, with common adverse effects including headache and scalp discomfort; rare serious effects include seizures and treatment‐emergent mania [5–7]. Although psychosis is not a typical adverse outcome, case reports describe emergent mania or psychotic symptoms in susceptible individuals, supporting plausibility in rare circumstances. Mechanistically, TMS may influence dopaminergic and glutamatergic transmission in cortico‐striatal circuits, which could theoretically contribute to aberrant salience, paranoia, or perceptual disturbances in vulnerable patients [16, 17]. Thus, in this case, TMS may have contributed to symptom escalation against a background of inflammatory stress and substance exposure.

### 3.3. The Long Shadows of Trauma and Familial Psychiatric History

Having a family history of mental illness and past traumatic experiences gives a compelling illustration of our patient’s descent into psychosis, influencing his mental health. Both his parents suffered from depression, and his father attempted suicide. In addition to this, his paternal grandfather also completed suicide by hanging himself, which predisposes our patient to a solid genetic component, making him vulnerable to mental health diseases. It has been found in generational family studies that the risk of psychopathology for offspring is higher in the previous three generations than in the two previous generations affected with depression [18]. This patient was also sexually abused by some boys in his neighborhood during his formative period, compounding his symptoms of depression and anxiety. Systematic review and meta‐analysis also reveal an increased risk for lifetime diagnosis of multiple psychiatric disorders like depression, anxiety, posttraumatic stress disorder (PTSD), and psychotic disorders in patients with a childhood history of sexual abuse [19–21]. He was unable to complete schooling traditionally due to the symptoms of depression and anxiety, leading to homeschooling and eventually social isolation, which affected his mental health. The retirement of his long‐term psychiatrist, who was his stabilizing factor for 27 years, coupled with his father’s death, precipitated his condition. Hence, this case emphasizes multifactorial interactions between environmental, genetic, and psychosocial factors in the onset of psychotic symptoms, determining the necessity for new and holistic treatment strategies.

### 3.4. Legal Stress, Shame, and Psychopathology

The patient’s legal investigation appears to have functioned as an extreme psychosocial stressor, amplifying shame, fear, hypervigilance, and rumination. Such stress may potentiate psychotic interpretations and exacerbate suicidal risk. This aligns with literature emphasizing the complex emotional and cognitive correlates of problematic pornography use and distress [22, 23], though causality cannot be inferred.

### 3.5. Pulmonary Complications and the Interplay With Psychiatric Symptoms

The intersection of mental health and inflammatory diseases is complex. Studies have shown that patients with pulmonary diseases, such as pneumonia or acute respiratory distress syndrome, and hospitalized for the same are prone to psychological disorders such as anxiety, depression, and psychosis. They suggest that these psychiatric conditions can be exacerbated by systemic inflammation and similar infections due to increased levels of proinflammatory cytokines [10, 11]. The reason for having such severe pneumonia associated with lung abscess may be due to poor living conditions, unemployment, and picking of synthetic cannabis packets from the ground to get high, leading to his mental health condition post‐infection.

### 3.6. Challenges in Treating Refractory Psychosis Symptoms With Standard Antipsychotics: Navigating the Storm

Individuals with complex psychiatric histories, like this patient, usually lack response to standard antipsychotic treatments, amplifying the challenges in managing refractory psychosis. This patient continued to experience severe psychiatric symptoms such as delusions, thought insertion, and broadcast despite treatment with potent first‐line antipsychotics such as haloperidol and risperidone, and a trial of clozapine, as it is considered the gold standard for treatment‐resistant psychotic patients and for effects. Several studies show that clozapine seems to be associated with substantially decreased mortality as well as risk of suicide among all antipsychotics, but restrictions on the use of clozapine should be reassessed [24–26]. A previous history of chronic cannabis use disorder is another risk factor contributing to psychotic symptoms [27, 28]. Long‐term cannabis use is linked to increased thoughts of suicide, risk of developing social anxiety disorder, and poor performance on memory tasks [27]. Another study revealed that chronic use of amitriptyline in adolescents and young adults may increase the risk of suicidal ideation and behavior [29]. It may be possible that all these contributing factors interfere with the effectiveness of TMS and treatment with antipsychotics. There is a need for individualized treatment plans, including psychotherapy and supportive housing, to address the multifactorial causation in this patient.

### 3.7. Unwilling the Mind: Psychological Testing and Insights

The patient was referred by his psychiatric treatment team for psychological assessment to provide information for a differential diagnosis. He was seen for 2.5 h for a clinical interview and administration of psychological measures (Minnesota Multiphasic Personality Inventory ‐ Third Edition (MMPI‐3) and Thematic Apperception Test (TAT) [30, 31]. He reported a history of sexual trauma in childhood, with alterations in mood and cognition and distress related to memories and reminders of the trauma, as well as a long history of recurrent Major Depressive Disorder, with episodes beginning in adolescence. He reported intrusive and unwanted thoughts that are ego‐dystonic and distressing, causing marked distress and discomfort, as well as efforts to manage anxiety through self‐talk to mitigate negative outcomes.

In this assessment, his thought process was organized and linear, with the endorsement of the unusual belief that others could hear his intrusive thoughts and follow him or act against him in some way. His MMPI‐3 reflected: struggles with negative emotional experiences, including anxiety, stress, and fear; demoralization and suicidality; suspiciousness with persecutory delusions; abnormal perceptual experiences, including hearing strange things; and significant substance use. His TAT stories were linear, well‐organized, and detailed; they suggested strong avoidance of negative affect and cognition. It appears likely that his abnormal perceptual experiences and persecutory beliefs relate to his significant anxiety and avoidance of negative emotions; however, the patient also reported significant substance use, with recent use of synthetic marijuana, and urine toxicology at admission that was positive for amphetamine. It is unclear the degree to which his abnormal perceptions and odd beliefs are related to his substance use.

The assessment concluded that his psychiatric presentation is suggestive of the following diagnoses: Other Trauma‐ and Stress‐related Disorder; Major Depressive Disorder, recurrent; Obsessive‐Compulsive Disorder with delusional beliefs; and Synthetic Marijuana (Spike) Use Disorder. The patient’s suicidality occurred in the context of intolerable ego‐dystonic thought broadcasting experiences and severe anxiety/shame. Contemporary perspectives describe suicidality in psychosis as multidimensional and heterogeneous, with important subtypes that may be influenced by substance exposure and distinct psychological drivers (e.g., defeat, entrapment, shame, fear). Clinically, this supports targeted assessment beyond symptom counting—evaluating affective distress, shame, impulsivity, substance effects, and perceived loss of control.

### 3.8. Pharmacology and Treatment Resistance: Interdisciplinary Implications

The limited response to multiple antipsychotic trials, including clozapine, raises questions about whether symptoms were driven by classic dopamine‐mediated psychosis versus anxiety/obsessional phenomena, trauma‐driven interpretations, and/or substance‐related neurotoxicity. From a pharmacology perspective, partial benefit from lorazepam may reflect anxiety‐driven amplification of intrusive experiences rather than psychosis alone. Interdisciplinary care integrating neuromodulation science, trauma‐focused psychotherapy, and substance‐use treatment may be essential in similar presentations.

### 3.9. Differential Diagnosis Considerations

Given the patient’s trauma history and intrusive ego‐dystonic phenomena, the differential diagnosis includes:

• PTSD with dissociative/psychotic‐like features (trauma‐related intrusions and hypervigilant interpretations).
• OCD with absent insight/delusional beliefs (intrusive thoughts misattributed as externally accessible, with associated compulsive safety behaviors).
• Substance/medication‐induced psychotic disorder (synthetic cannabinoids, stimulants; chronic cannabis).
• Primary psychotic disorder/late‐onset schizophrenia‐spectrum disorder (less likely given late onset and prominent anxiety/ego‐dystonic distress, but cannot be excluded).

Psychological testing (MMPI‐3, TAT) supported prominent anxiety, suspiciousness, unusual beliefs about others hearing thoughts, and substance‐use burden, with diagnostic impressions including trauma‐related disorder, recurrent MDD, OCD with delusional beliefs, and synthetic cannabinoid use disorder.

### 3.10. Clinical Implications

1. Do not assume single‐cause attribution (e.g., ‘TMS‐induced psychosis’) when new‐onset psychosis appears; instead, assess for medical inflammation, substance exposure, trauma‐related vulnerability, and acute psychosocial stressors.
2. Monitoring during TMS: in patients with complex trauma, heavy substance use, or severe anxiety, implement structured monitoring for emergent paranoia, hallucinations, and ego‐dystonic FRS‐like experiences, with rapid reassessment if symptoms arise.
3. Suicide risk assessment must be multidimensional: assess shame/defeat, perceived loss of control, substance‐related intoxication/withdrawal states, and fear‐driven avoidance, not solely command hallucinations or depressive severity.

### 3.11. Research and Future Directions

We recommend prospective or registry‐based surveillance of TMS safety outcomes in higher‐risk populations (complex trauma, significant substance use) to quantify the incidence of emergent psychotic symptoms and suicide‐related outcomes. Development of consensus monitoring checklists (brief psychosis screen + substance screen + suicide subtype‐informed assessment) during neuromodulation courses. Longitudinal studies examining whether inflammatory states (e.g., post‐severe infection) increase vulnerability to neuromodulation‐related neuropsychiatric adverse events.

## 4. Conclusion

This case illustrates the sudden emergence of FRS‐like symptoms and severe suicidality in a patient with chronic depression and complex trauma following a period of severe medical illness, substance exposure, acute legal stress, and TMS. The clinical picture supports a biopsychosocial convergence model in which these intersecting factors may disrupt cerebral homeostasis and precipitate psychosis‐like experiences and suicide attempts. While TMS may have contributed as a precipitating factor, the overall presentation is unlikely to be attributable to a single cause.

Clinicians should maintain vigilance for emergent psychosis and multidimensional suicide risk during neuromodulation, especially in patients with trauma histories, heavy substance exposure, and major psychosocial stress. Further research—including registries and longitudinal safety studies—is needed to clarify risk factors and monitoring guidelines.

## Funding

This study was supported by the State University of New York Upstate Medical University, which will cover the article processing charges through a designated departmental budget.

## Consent

Written informed consent was obtained from the patient for publication of this case report and all accompanying clinical information. Identifying details have been omitted to ensure patient confidentiality.

## Conflicts of Interest

The authors declare no conflicts of interest.

## Data Availability

The data that support the findings of this study are available from the corresponding author upon reasonable request.
